# All-Cause And Cardiovascular Mortality in Older Adults With Multimorbidity : 1999-2018 National Survey

**DOI:** 10.1093/geroni/igaf122.3356

**Published:** 2025-12-31

**Authors:** Chitchanok Benjasirisan, Arum Lim, Asma Rayani, Soo Hyun Kim, Cheryl Himmelfarb, Patricia Davidson, Binu Koirala

**Affiliations:** Johns Hopkins University, Baltimore, Maryland, United States; Johns Hopkins University School of Nursing, Baltimore, Maryland, United States; Johns Hopkins University, Baltimore, Maryland, United States; Johns Hopkins University, Baltimore, Maryland, United States; Johns Hopkins University, Baltimore, Maryland, United States; University of New South Wales Sydney, Kensington, New South Wales, Australia; Johns Hopkins University, Baltimore, Maryland, United States

## Abstract

Multimorbidity, the presence of two or more chronic conditions, is associated with premature mortality and decreasing life expectancy. Cardiovascular disease (CVD) remains a leading cause of death in the U.S. and is often final pathway to death among people living with multimorbidity. This study examined the association between multimorbidity and mortality, specifically CVD and all-cause mortality, among U.S. adults aged 50 years of age and older. Data from the 1999-2018 National Health Interview Survey linked with the National Death Index were analyzed. Primary outcomes were all-cause and CVD mortality (due to heart disease and cerebrovascular disease) years, measured from the interview to death or December 31, 2019. Cox regression models assessed mortality risk differences, adjusting for age, sex, marital status, poverty-income ratio, and education. Among 264,400 adults (39.02% with 0-1 chronic conditions; 60.98% with ≥2 conditions), the median follow-up was 7 years (IQR: 4-12). All-cause mortality was 30.65 per 1,000 person-years and CVD mortality was 9.91 per 1,000 person-years. Multimorbidity was linked to higher mortality rate (all-cause: 38.66 vs. 19.75 and CVD: 12.79 vs. 5.70, per 1,000 person-years). Fully adjusted models showed a 43% higher all-cause mortality risk (95% CI: 1.40-1.46) and 59% higher CVD mortality risk (95% CI: 1.52-1.65) for those with multimorbidity. Additionally, each additional comorbidity was associated with an increased risk in all-cause (aHR:1.06; 95% CI: 1.04–1.09) and CVD mortality (aHR: 1.14; 95% CI: 1.10–1.18). Integrated models of care considering multidisciplinary management is crucial to improve health outcomes and mitigate mortality risk among older adults with multimorbidity.

